# Repeated plasma p‐tau217 measurements to monitor clinical progression heterogeneity

**DOI:** 10.1002/alz.70319

**Published:** 2025-05-29

**Authors:** Bjørn‐Eivind Kirsebom, Fernando Gonzalez‐Ortiz, Sinthujah Vigneswaran, Geir Bråthen, Ragnhild Eide Skogseth, Berglind Gísladóttir, Peter Harrison, Jonas Alexander Jarholm, Lene Pålhaugen, Arvid Rongve, Per Selnes, Betty Tjims, Michael Turton, Argonde C. Van Harten, Knut Waterloo, Henrik Zetterberg, Tormod Fladby, Kaj Blennow

**Affiliations:** ^1^ Department of Neurology University Hospital of North Norway Tromsø Norway; ^2^ Department of Psychology Faculty of Health Sciences The Arctic University of Norway Tromsø Norway; ^3^ Department of Neurology Akershus University Hospital Lørenskog Norway; ^4^ Institute of Neuroscience and Physiology University of Gothenburg Mölndal Sweden; ^5^ Clinical Neurochemistry Lab Sahlgrenska University Hospital Mölndal Sweden; ^6^ Department of diagnostics Neurocode USA Inc Bellingham Washington USA; ^7^ Neurochemistry Laboratory Department of Clinical Chemistry Amsterdam Neuroscience Vrije Universiteit Amsterdam Amsterdam UMC Amsterdam The Netherlands; ^8^ Alzheimer Center Amsterdam Neurology Department Vrije Universiteit Amsterdam Amsterdam UMC Location VUmc Amsterdam The Netherlands; ^9^ Amsterdam Neuroscience, Neurodegeneration Amsterdam The Netherlands; ^10^ Department of Neurology and Clinical Neurophysiology University Hospital of Trondheim Trondheim Norway; ^11^ Department of Neuromedicine and Movement Science Faculty of Medicine and Health Sciences Norwegian University of Science and Technology Trondheim Norway; ^12^ Department of Geriatric Medicine Haraldsplass Deaconess Hospital Bergen Norway; ^13^ Department of Clinical Sciences Faculty of Medicine University of Bergen Bergen Norway; ^14^ Bioventix Plc, 7 Romans Business Park, East Street Farnham Surrey UK; ^15^ Institute for Clinical Medicine Campus Ahus University of Oslo Oslo Norway; ^16^ Department of Neuropsychology Haugesund Hospital Haugesund Norway; ^17^ Department of Clinical Medicine (K1) University of Bergen Bergen Norway; ^18^ Department of Research Akershus University Hospital Lørenskog Norway; ^19^ UK Dementia Research Institute at UCL London UK; ^20^ Department of Neurodegenerative Disease UCL Institute of Neurology Queen Square London UK; ^21^ Hong Kong Center for Neurodegenerative Diseases Clear Water Bay Hong Kong China; ^22^ Wisconsin Alzheimer's Disease Research Center University of Wisconsin School of Medicine and Public Health University of Wisconsin‐Madison Madison Wisconsin USA; ^23^ Paris Brain Institute ICM, Pitié‐Salpêtrière Hospital Sorbonne University Paris France; ^24^ Neurodegenerative Disorder Research Center Division of Life Sciences and Medicine, and Department of Neurology Institute on Aging and Brain Disorders University of Science and Technology of China and First Affiliated Hospital of USTC Hefei P.R. China

**Keywords:** Alzheimer's disease, amyloid, cerebrospinal fluid, clinical heterogeneity, clinical progression, clinical trials, plasma, p‐tau217

## Abstract

**INTRODUCTION:**

Heterogeneity of clinical progression in Alzheimer's disease (AD) complicates the assessment of disease progression and treatment effects in trials. This study evaluates the potential of plasma phosphorylated tau‐217 (p‐tau217) to capture this heterogeneity.

**METHODS:**

We used k‐means clustering to analyze cognitive trajectories in amyloid beta –positive (Aβ+) cognitively normal (CN) and mild cognitive impairment (MCI) participants from two independent cohorts. Cohort 1 included 186 participants (71 CN, 115 MCI; 507 observations) and Cohort 2 included 207 participants (64 CN, 144 MCI; 781 observations), both with up to 10 years of follow‐up.

**RESULTS:**

Three progression clusters emerged in both cohorts: stable cognition, slow decline, and rapid decline—each including cases initially classified as CN or MCI. Baseline plasma p‐tau217 was linked to progression risk in both cohorts, whereas longitudinal increases in Cohort 1 were steepest in rapid decliners.

**DISCUSSION:**

Plasma p‐tau217 may aid in capturing clinical heterogeneity and support stratification and monitoring of disease progression in clinical trials.

**Highlights:**

- k‐Means found stable, slow, and rapid cognitive decline clusters in amyloid beta–positive (Aβ+) cases.
- Higher baseline plasma phosphorylated tau‐217 (p‐tau217) levels predicted faster cognitive decline.
- Longitudinal increases in plasma p‐tau217 were steepest in rapid decliners.
- Plasma p‐tau217 tracks clinical progression heterogeneity in Aβ+ cases.
- Cognitive stage and amyloid alone may miss severity and risk in early‐stage Alzheimer's disease.

**Abbreviations**
AD: Alzheimer's disease
Aβ: amyloid beta
CI: confidence interval
CN: cognitively normal
CSF: cerebrospinal fluid
LLM: Linear Mixed Model
MCI: mild cognitive impairment
p‐tau: phospho‐tau
SD: standard deviation

## 1 BACKGROUND

In recent years, Alzheimer's disease (AD) treatment has entered a transformative era with the development of monoclonal antibody (mAb) disease‐modifying therapies targeting amyloid beta (Aβ).^1^, ^2^ Although effective in clearing Aβ deposits and reducing biomarkers such as phosphorylated tau (p‐tau), these treatments have shown only modest effects of slowing cognitive decline over 18‐month trials.^3^, ^4^ This has sparked a debate concerning their clinical benefits versus costs and side effects.^5^ Heterogeneity in clinical severity and progression may be linked to various sources, including AD pathological burden and genetic predisposition,^6^ and co‐pathologies such as vascular pathology, Lewy body pathology, and TAR DNA‐binding Protein (TDP‐43) inclusions.^7^

The donanemab Phase 3 study stratified study participants by tau positron emission tomography (tau‐PET) tau pathology burden, showing greater slowing of progression in individuals with lower tau burden.^2^ Arguably, this captures an important element of the heterogeneity of clinical progression, as tau‐tangle burden reflects AD severity, with higher burden increasing the likelihood of clinical progression.^8^ Large anti‐amyloid mAb trials targeting preclinical AD cases (biomarker‐positive cognitively normal individuals) are ongoing.^9^, ^10^ In such trials, incorporating biomarkers capable of capturing the heterogeneity of clinical progression is crucial, since not all participants with Aβ deposits (Aβ+) will develop mild cognitive impairment (MCI) or dementia in their lifetimes.^11^ Treating patients earlier in the disease course may enhance the efficacy of anti‐amyloid therapies in slowing progression,^12^ but determining which patients are most likely to benefit is essential for balancing risks and benefits.

Conventional neuropsychological criteria are often used to determine clinical stage.^13^, ^14^ However, there is considerable within‐group variability in neuropsychological test performance among individuals in both the preclinical and MCI stages, with some cases falling near the threshold between cognitive normality and impairment.^14^ Moreover, lower normative performance of neuropsychological tests not only captures departures from the mean due to neurological disease, but also reflects individual differences in cognitive performance,^15^, ^16^ and premorbid intelligence levels associated with cognitive reserve mechanisms.^17^ Thus individual rates of cognitive change may be a more robust measure of heterogeneity.^18^

Plasma p‐tau217 has been validated as a sensitive marker for AD pathophysiology. We and other groups have reported high diagnostic accuracies of plasma p‐tau217 in AD.^19^, ^20^, ^21^ It has been endorsed for use as a Core 1 AD biomarker by the Alzheimer's Association (AA)^22^ and used as a part of screening for clinical trials.^23^ Beyond its diagnostic utility, higher plasma p‐tau217 levels are linked to greater tau‐tangle burden,^24^ increased clinical severity in cross‐sectional studies,^19^ faster clinical progression,^19^, ^25^, ^26^ and longitudinal increases that reflect both biological and clinical severity.^21^, ^27^ As with cognitive tests, within‐group variations in plasma p‐tau‐217 concentrations are seen in both cognitively normal and MCI cases^19^, ^21^, ^28^ and could in part reflect disease severity and an individual's risk for future clinical progression. The longitudinal dynamics of plasma p‐tau217 levels could be a way to address this variation, and repeated measurements of this marker could be an accessible tool to monitor both disease progression and treatment effect.^27^

In this study, we aim to (1) assess heterogeneity of clinical progression in cerebrospinal fluid (CSF) Aβ‐positive (Aβ+) cognitively normal (CN), subjective cognitive decline (SCD), and MCI individuals using data‐driven methods in two independent cohorts with up to 10 years of follow‐up; and (2) investigate whether cross‐sectional and longitudinal changes in plasma p‐tau217 align with the heterogeneity of clinical progression. We hypothesize that assessing individual rates of change in Aβ+ participants will help classify individuals according to their clinical progression risk, including non‐progressors.

**RESEARCH IN CONTEXT**

1. **Systematic review**: We reviewed the existing literature on plasma phosphorylated tau‐217 (p‐tau217) as a biomarker for Alzheimer's disease (AD) and its potential role in tracking disease progression. Prior studies have established plasma p‐tau217 as a sensitive marker of AD pathology and its association with clinical progression. However, its ability to capture individual differences in disease trajectories and the heterogeneity of clinical progression remain underexplored.
2. **Interpretation**: Our findings demonstrate that plasma p‐tau217 tracks the heterogeneity of clinical progression in amyloid‐positive cognitively normal and individuals with mild cognitive impairment. Higher baseline levels and steeper longitudinal increases were associated with faster cognitive decline, suggesting its potential for stratifying patients and monitoring disease progression.
3. **Future directions**: Further research should validate these findings in larger cohorts, explore co‐pathologies that influence progression, and assess the utility of plasma p‐tau217 in monitoring treatment response. In addition, integrating p‐tau217 with other biomarkers may enhance the predictive accuracy for disease progression and therapeutic outcomes.

## 2 METHODS

### 2.1 Cohort 1

The Norwegian multicenter Dementia Disease Initiation (DDI) study cohort includes individuals 40–80 years of age, recruited from memory clinics and local advertisements across Norway. The cohort comprises cognitively normal participants, with or without SCD, and individuals with MCI. Participants undergo standardized diagnostic workups, including medical history, neurological examination, neuropsychological testing, and collection of CSF and blood samples, with reassessments approximately every 2 years. Detailed cohort characteristics and diagnostic criteria have been described previously.^29^ However, in the present study, we applied an actuarial definition of MCI based solely on neuropsychological criteria, without requiring the presence of cognitive symptoms, as described in our previous work.^30^ From this cohort, a total of 186 individuals with pathological CSF Aβ42/40 ratios were included, comprising 71 CN/SCD cases of whom 17 (23.9%) had SCD and 54 (76.1%) did not, and 115 cases with MCI. Among CN/SCD cases, 36 (50.7%) were recruited through advertisements and 35 (49.3%) from memory clinics. Among MCI cases, 41 (35.7%) were recruited via advertisements and 74 (64.3%) from memory clinics. In addition, 19 spouses of memory clinic participants, originally recruited as healthy controls, were found to have established amyloid pathology. Of these, seven had normal cognition with no cognitive symptoms, three had SCD, and nine fulfilled our MCI criteria. All had available neuropsychological follow‐up assessments (a total of 507 observations, between 0.77 and 10.56 years from baseline; mean = 3.49, SD = 1.90). The DDI study was approved by the Regional Committees for Medical and Health Research Ethics in Norway and conducted in line with the guidelines provided by the Declaration of Helsinki and the Norwegian Health and Research act. All participants gave written informed consent before participating in the study.

### 2.2 Cohort 2

The Amsterdam Dementia Cohort (ADC) comprises individuals who visited the Alzheimer Center of the Amsterdam UMC, location VU University Medical Center (VUmc) in Amsterdam, a tertiary memory clinic.^31^ Individuals referred to the Alzheimer Center Amsterdam Center Amsterdam receive standardized diagnostic workup, including neurological investigation by a neurologist, neuropsychological testing, assessment of vital functions, and CSF draw. Diagnoses were made based on international consensus criteria^32^, ^33^, ^34^ during a weekly multidisciplinary meeting. Individuals were included when they had a diagnosis of SCD, considered as cognitively normal, or MCI due to AD based on CSF (see below) and available neuropsychological follow‐up assessments. From this cohort, a total of 207 cases (63 SCD and 144 MCI) with pathological CSF Aβ1‐42 were selected. All had available neuropsychological follow‐up assessments (a total of 781 observations, between 0.04 and 9.61 years from baseline; mean = 2.91, SD = 1.85). The ADC study was approved by the ethics committee of the Amsterdam UMC and the Biobank Research Ethics Committee of the Amsterdam UMC (location VUmc). All patients gave written informed consent, and the study was conducted in accordance with the guidelines provided by the Declaration of Helsinki.

### 2.3 CSF AD biomarkers

In Cohort 1, the QuickPlex SQ 120 system from Meso Scale Discovery (MSD; MD, USA) was used to measure Aβ1‐42 and Aβ1‐40 in a multiplex setup using V‐plex Ab Peptide Panel 1 (6E10) kit (K15200E‐1). The cutoff for CSF Aβ42/40 ratio of ≤ 0.077 was determined following receiver‐operating characteristic (ROC) curve analysis using visual read of [18F]‐flutemetamol PET scans as the standard of truth.^35^ In Cohort 2, Aβ1‐42 concentration was measured using an INNOTEST enzyme‐linked immunosorbent assay (ELISA) from Fujirebio (Ghent, Belgium). The previously published cutoff of <813 pg/mL was used to determine the presence or absence of cerebral Aβ pathology.^36^

### 2.4 Plasma p‐tau217 measurements

In Cohort 1, p‐tau217 levels were measured using the University of Gothenburg in‐house assay,^37^ conducted on the Simoa HD‐X platform. The assay utilized a 1:2 dilution factor. To monitor signal variability within and between analytical runs, three internal quality control samples were analyzed at both the start and end of each run. A total of *n* = 175/186 (94.1%) cases had available plasma p‐tau217 coinciding with baseline cognitive measurements. In addition, *n* = 185 (99.46%) of our sample had available repeated plasma measurements for our clusters (438 observations between 0.77 and 9.66 years from baseline; mean = 3.75 years, SD = 1.74). In Cohort 2, plasma p‐tau217 concentration was measured using Simoa HD‐X platform and the p‐tau217 kit (Janssen Sciences Ireland UC, a Johnson & Johnson company) according to the manufacturer's protocol. A subset of cases (79/207, 38%) had available plasma p‐tau217 measured at baseline.

### 2.5 Cognitive composite scores

We assessed longitudinal changes in neuropsychological test performance across the available tests in Cohort 1^29^ using linear mixed‐effects models (LMMs) with random intercepts for subjects and random slopes for time. Marginal *R* ^2^ values, representing the proportion of variance in cognitive test performance explained by time, were computed using the “performance” R package. Among the tested measures, the Controlled Oral Word Association Test (COWAT) and Visual Object and Space Perception Battery (VOSP) Silhouettes tests explained 0.3% and 0.4% of the variance, respectively, whereas the Trail Making Test Part B (TMT‐B) and Consortium to Establish a Registry for Alzheimer's Disease (CERAD) memory recall test explained 2.5% and 2.9%, respectively. Based on these analyses, TMT‐B and CERAD memory recall were selected for inclusion in the cognitive composite score, as they exhibited the most pronounced changes over time in participants with CSF Aβ+ status (Table S1). To create a cognitive composite score, we used the established methods.^38^ The CERAD memory recall test was standardized to a score between 0 and 1 using the following formula: (raw score—minimum possible score)/(maximum possible score—minimum possible score). Because higher TMT‐B scores indicate worse performance, the scores were standardized to a score between 0 and 1 using the formula: (maximum possible score—raw score)/(maximum possible score—minimum possible score), so that higher standardized scores reflect better performance. These scores were summed and averaged to compute a 0–1 standardized composite score. The composite model explained 4.8% of the variance in cognitive change (Table S1), indicating that combining TMT‐B and CERAD memory recall improved the overall prediction of cognitive decline. In all models, conditional *R* ^2^ values (representing variance explained by both fixed and random effects) ranged from 74% to 85%, highlighting the substantial contribution of subject‐specific variations in intercept and slope. A similar approach was applied to Cohort 2, where the Rey Auditory Verbal Learning Test (RAVLT) delayed memory recall and TMT‐B were selected based on the same criteria. These scores were standardized and combined using the same method as described above to create a composite cognitive score for Cohort 2.

### 2.6 Statistical analyses

All analyses were conducted in RStudio (R version 4.3.2). Baseline between‐group comparisons of continuous variables (e.g., age, years of education, CSF, and plasma biomarkers) were performed using linear regression analyses. For dichotomous variables (i.e., sex) chi‐square analyses were used. Longitudinal trajectories of cognitive composite scores were modeled using LMMs with random intercepts for subjects and random slopes for time in both cohorts. For our main models, we opted to not include demographics (age, sex or educational level). Although such variables influence cognitive performance in normative contexts,^15^, ^16^ they are also associated with AD risk,^39^, ^40^ and all our participants had established amyloid pathology. Instead, the individual random slopes (i.e., individual trajectories over time) were extracted, regardless of demographics, and clustered using k‐means clustering with Euclidean distance. The optimal number of clusters was determined visually via elbow plots (Figure S1A,B).

Between‐cluster comparisons of baseline plasma p‐tau217 levels were then conducted in both cohorts using linear regression, and post hoc comparisons were conducted with false‐discovery rate (FDR) adjustments within each cohort. In addition, the mean fold change for each cognitive decline cluster with stable cognition as the reference group was computed in both cohorts. Finally, an LMM model was fitted to assess longitudinal changes in plasma p‐tau217 levels in Cohort 1, with random slopes for time included in this model. As a sensitivity analysis, we re‐ran the LMMs including demographics (age at baseline, sex, and years of education) as covariates when estimating individual slopes. These adjusted slopes were then clustered using the same k‐means approach. Agreement between cluster solutions derived with and without demographic adjustment was assessed using Cohen's kappa.

## 3 RESULTS

### 3.1 Cohort characteristics

Cohort 1 participants were older (mean = 67.99, SD = 7.50) and more educated (mean = 13.66, SD = 3.12) than Cohort 2 (mean = 65.89, SD = 6.75, *p* < 0.001; mean = 12.34, SD = 3.12, *p* < 0.001), but sex distribution was comparable (Cohort 1: 53.2% female; Cohort 2: 48.3% female; *χ* ^2^ = 0.76, *p* = 0.38). No differences in age, education, or sex distributions were observed between Aβ+ CN and Aβ+ MCI groups in either cohort. In Cohort 1, CSF Aβ42/40 ratios did not differ between groups, but in both cohorts, Aβ+ MCI participants had lower Aβ1‐42 levels than Aβ+ CN participants (Cohort 1, *p* < 0.05; Cohort 2, *p* < 0.01; see Table 1).

**TABLE 1:** Between‐group comparisons of demographics, cognitive measures, and CSF Aβ and plasma p‐tau217 markers in the Dementia Disease Initiation (DDI) cohort and Amsterdam Dementia Cohort (ADC).

	Cohort 1: Dementia Disease Initiation		
	CN/SCD Aβ+ *n* = 71	MCI Aβ+ *n* = 115	Total *n* = 186
**Age,** mean (SD)	67.54 (7.50)	68.27^n.s.^ (7.52)	67.99 (7.50)
**Years of education,** mean (SD)	13.68 (3.03)	13.65^n.s.^ (3.19)	13.66 (3.12)
**Female,** n (%)	41 (57.7%)	58^n.s.^ (51.4%)	99 (53.2%)
^a^ **Aβ42/40 ratio, mean (SD)**	0.054 (0.013)	0.051^n.s.^ (0.012)	0.052 (0.012)
^a^ **Aβ1‐42, pg/mL,** mean (SD)	413.42 (158.62)	368.65^*^ (116.93)	384.80 (135.03)
^b^ **Plasma p‐tau217, pg/mL,** nean (SD) [n]	2.82 (1.24) [66]	3.40^*^ (1.55) [109]	3.18 (1.46)
**TMT‐B,** mean (SD)	84.79 (20.99)	155.75^***^ (115.09)	128.66 (97.59)
**CERAD memory recall,** mean (SD)	7.07 (1.91)	3.56^***^ (2.41)	4.90 (2.81)
**Cognitive composite,** mean (SD)	0.66 (0.11)	0.42^***^ (0.15)	0.51 (0.18)
^e^ **Observations over time,** n (%)	205 (40.4%)	302 (59.6%)	507 (100%)
^e^ **Years since baseline,** mean (SD)	3.80 (1.95)	3.26 (1.84)	3.49 (1.90)

	Cohort 2: Amsterdam Dementia Cohort		
	SCD Aβ+ *n* = 63	MCI Aβ+ *n* = 144	Total *n* = 207
**Age,** mean (SD)	65.18 (6.44)	66.20^n.s.^ (6.88)	65.89 (6.75)
**Years of education,** mean (SD)	12.78 (3.17)	12.15^n.s.^ (3.09)	12.34 (3.12)
**Female,** n (%)	36 (57.1%)	64^n.s.^ (44.4%)	100 (48.3)
^c^ **Aβ1‐42,** mean (SD)	659.36 (100.52)	621.24^**^ (116.19)	632.84 (112.79)
^d^ **Plasma p‐tau217, pg/mL,** mean (SD) [n]	0.07 (0.04)	0.08^n.s.^ (0.04)	0.08 (0.04)
**TMT‐B,** mean (SD)	87.62 (32.42)	121.53^***^ (59.64)	111.21 (55.04)
**RAVLT memory recall,** mean (SD)	7.49 (2.87)	3.12^***^ (2.44)	4.45 (3.27)
**Cognitive composite,** mean (SD)	0.59 (0.13)	0.39^***^ (0.12)	0.45 (0.15)
^e^ **Observations over time,** n (%)	230 (29.4%)	551 (70.6%)	781 (100%)
^e^ **Years since baseline,** mean (SD)	3.20 (2.10)	2.79 (1.72)	2.91 (1.85)

*Note*: Cognitive composite (Trail Making Test, Part B [TMT‐B] + memory recall on a 0–1 scale).

Abbreviations: %, percentage; Aβ± , positive or negative cerebrospinal fluid (CSF) marker for amyloid beta (Aβ) plaques; CN, cognitively normal; MCI, mild cognitive Impairment; n, number of cases; phosphorylated tau 217 (p‐tau217);

n.s. = non‐significant; CERAD, consortium to establish a registry for alzheimer's disease; RAVLT, rey auditory verbal learning test; SCD, subjective cognitive decline; SD, standard deviation.

^a^ Mesoscale discovery Aβ1 assay.

^b^ University of Gothenburg Simoa p‐tau217 assay.

^c^ Innotest Aβ1‐42 assay.

^d^ Janssen Simoa p‐tau217 assay.

^e^ No statistical comparison performed.

* < 0.05.

** < 0.01.

*** < 0.001.

### 3.2 Clusters of cognitive change

**FIGURE 1 alz70319-fig-0001:**
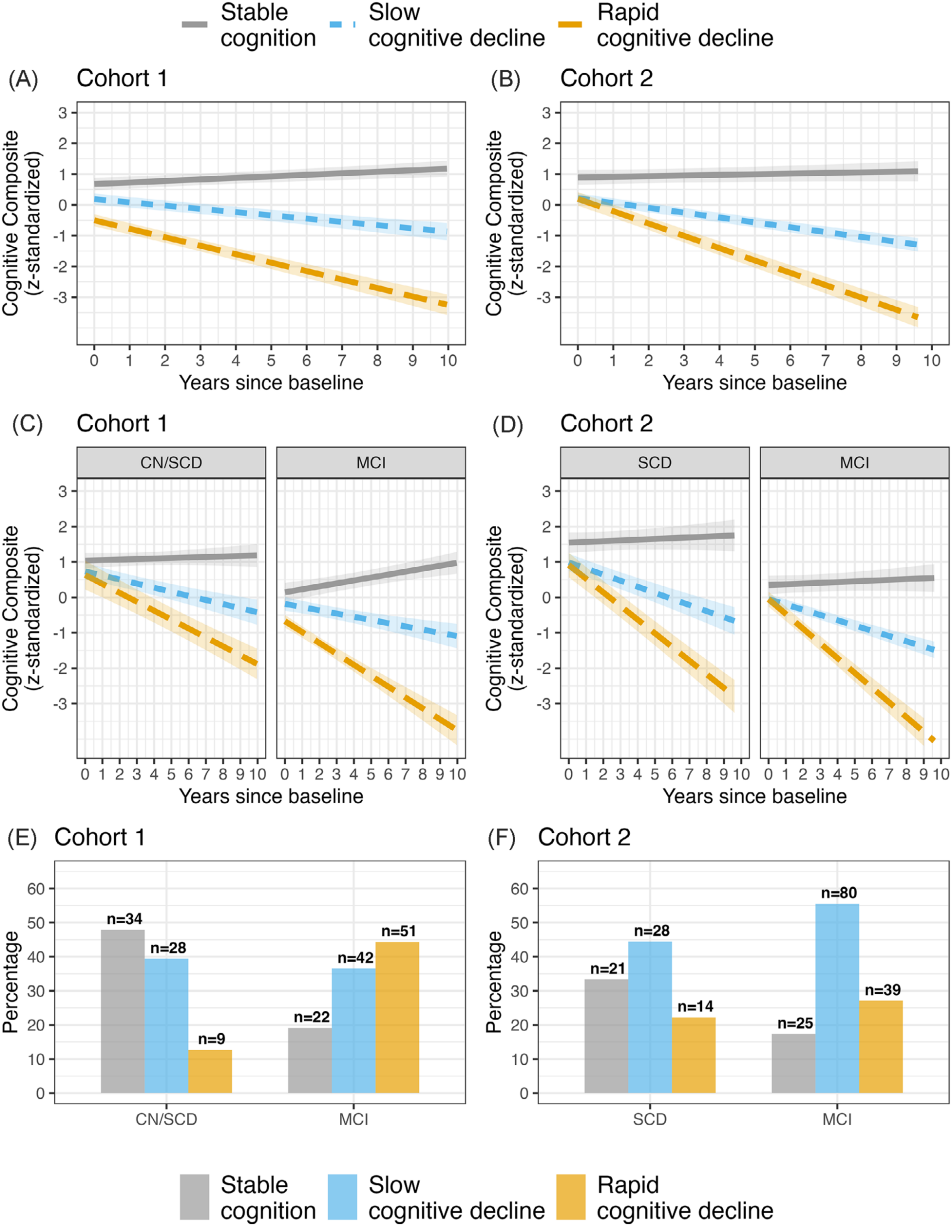
Illustration of the cognitive clusters derived from k‐means in both Cohort 1 and Cohort 2. (A) Cohort 1 and (B) Cohort 2 illustrate trajectories over time for each cluster. (C) Cohort 1 and (D) Cohort 2 illustrate trajectories over time for each cluster split by baseline cognitive stage (cognitively normal [CN]/subjective cognitive decline [SCD] or mild cognitive impairment [MCI]). Bands associated with each regression line illustrated 95% confidence intervals. (E) Cohort 1 and (F) Cohort 2 illustrate numbers and percentages of CN/SCD and MCI at baseline within each cluster.

Using k‐means clustering on cognitive change trajectories, three clusters were identified: stable cognition (Cohort 1: *n* = 56; Cohort 2: *n* = 46), slow cognitive decline (Cohort 1: *n* = 70; Cohort 2: *n* = 108), and rapid cognitive decline (Cohort 1: *n* = 60; Cohort 2: *n* = 53) (Figure 1A, B). All clusters included baseline Aβ+ CN and Aβ+ MCI cases, although cluster distributions varied, with more MCI cases in the slow and rapid decline clusters than in the stable cluster (Table 2, Figure 1E,F). Cognitive changes over time aligned with cluster membership, regardless of baseline CN or MCI diagnosis (Figure 1B,C). Worse baseline cognitive composite scores were observed in the slow and rapid decline clusters compared to the stable cluster in both Cohort 1 (*p* < 0.01, *p* < 0.001) and Cohort 2 (*p* < 0.001, *p* < 0.01), but no significant differences in age, education, or sex were found between clusters (Table 2). In Cohort 1, the slow and rapid decline clusters showed lower CSF Aβ1‐42 concentrations (*p* < 0.05) and Aβ42/40 ratios (*p* < 0.05, *p* < 0.01) compared to the stable cluster. In Cohort 2, no significant differences in CSF Aβ1‐42 concentrations were observed across clusters (Table 2).

**TABLE 2 alz70319-tbl-0002:** Between‐cluster comparisons of baseline demographics, follow‐up time, and CSF Aβ markers in the Dementia Disease Initiation (DDI) cohort and Amsterdam Dementia Cohort (ADC).

	Cohort 1: Dementia Disease Initiation			
	Stable cognition *n* = 56	Slow cognitive decline *n* = 70	Rapid cognitive decline *n* = 60	Slow versus rapid cognitive decline
**Age,** mean (SD)	66.25 (8.37)	68.03^n.s.^ (6.49)	69.57^n.s.^ (7.53)	^n.s.^
**Years of education,** mean (SD)	14.02 (3.18)	13.70^n.s.^ (3.25)	13.28^n.s.^ (2.92)	^n.s.^
**Female,** n (%)	26 (46.4)	44 (62.9)	29 (48.3)	^n.s.^
^a^ **Aβ42/40 ratio,** mean (SD)	0.057 (0.013)	0.051^*^ (0.012)	0.049^**^ (0.010)	^n.s.^
^a^ **Aβ1‐42,** mean (SD)	433.56 (143.44)	361.88^*^ (119.37)	360.42^*^ (128.46)	^n.s.^
^b^ **Plasma p‐tau217, pg/mL,** mean (SD) [n]	2.46 (1.22) [49]	3.13^**^ (1.34) [68]	3.84^***^ (1.51) [58]	^*^
**TMT‐B,** mean (SD)	115.5 (117.38)	124.33^n.s.^ (94.09)	146.00^**^ (78.56)	^n.s.^
**CERAD memory recall,** mean (SD)	6.46 (2.58)	5.16^*^ (2.50)	3.13^***^ (2.37)	^***^
**Cognitive composite,** mean (SD)	0.61 (0.16)	0.53^*^ (0.15)	0.40^***^ (0.16)	^***^
**CN/SCD,** n (%)	34 (60.7)	28 (40)	9 (15.0)	^***^
**MCI,** n (%)	22 (39.3)	42 (60)	51 (85)	
^e^ **Observations over time,** n (%)	169 (33.3)	181 (35.7)	157 (31.0)	
^e^ **Years since baseline,** mean (SD)	3.93 (1.96)	3.41 (1.87)	3.05 (1.78)	

	Cohort 2: Amsterdam Dementia Cohort			
	Stable cognition 46	Slow cognitive decline 108	Rapid cognitive decline 53	Slow versus rapid cognitive decline
**Age,** mean (SD)	64.65 (7.12)	66.13^n.s.^ (6.41)	66.47^n.s.^ (7.09)	^n.s.^
**Years of education,** mean (SD)	12.80 (3.10)	12.12^n.s.^ (3.06)	12.40^n.s.^ (3.27)	^n.s.^
**Female,** n (%)	20 (43.5%)	53 (49.1%)	27 (50.9)	^n.s.^
^c^ **Aβ1‐42,** mean (SD)	664.61 (99.25)	619.0^n.s.^ (117.39)	633.47^n.s.^ (110.58)	^n.s.^
^d^ **Plasma p‐tau217, pg/mL,** mean (SD) [n]	0.062 (0.041)	0.079^n.s.^ (0.045)	0.097^*^ (0.042)	^n.s.^
**TMT‐B,** mean (SD)	94.59 (40.87)	116.32^*^ (60.81)	115.23^*^ (51.32)	^n.s.^
**RAVLT memory recall,** mean (SD)	5.71 (3.89)	3.93^**^ (3.14)	4.34^n.s.^ (2.63)	^n.s.^
**Cognitive composite,** mean (SD)	0.52 (0.17)	0.43^***^ (0.15)	0.44^**^ (0.13)	^n.s.^
**CN/SCD,** n (%)	21 (45.6)	28 (25.9)	14 (26.4)	^*^
**MCI** n (%)	25 (54.3)	80 (74.1)	39 (73.6)	
^e^ **Observations over time,** n (%)	172 (22.0)	404 (51.7)	205 (26.2)	
^e^ **Years since baseline,** mean (SD)	3.02 (1.96)	2.99 (1.91)	2.66 (1.58)	

*Note*: Cognitive composite (Trail Making Test, Part B (TMT‐B) + memory recall on a 0–1 scale).

Abbreviations: %, percentage; CN, cognitively normal; MCI, mild cognitive Impairment; n, number of cases; n.s. = non‐significant; SCD, subjective cognitive decline; SD, standard deviation; CERAD, Consortium to Establish a Registry for Alzheimer's Disease; RAVLT, Rey Auditory Verbal Learning Test.

^a^ Mesoscale discovery Aβ assay.

^b^ University of Gothenburg Simoa p‐tau217 assay.

^c^ Innotest Aβ assay.

^d^ Janssen Simoa p‐tau217 assay.

^e^ No statistical comparison performed.

* < 0.05.

** < 0.01.

*** < 0.001 as compared with the stable cognition group, if not otherwise specified.

Our sensitivity analysis, which derived clusters of cognitive change trajectories while accounting for demographics (age at baseline, sex, and years of education), demonstrated almost perfect agreement with the original clusters derived without demographics in both cohort 1 (*k* = 0.951, *z* = 18.3, *p* < 0.001) and cohort 2 (*k* = 0.905, *z* = 18.0, *p* < 0.001). It is important to note that as in the original clustering, no significant differences in age, sex, or years of education were observed between clusters (see Figure S2 and Table S2), further supporting the robustness of the findings and indicating minimal influence of demographic factors on cluster composition.

### 3.3 Baseline plasma p‐tau217 differences between clusters

**FIGURE 2:**
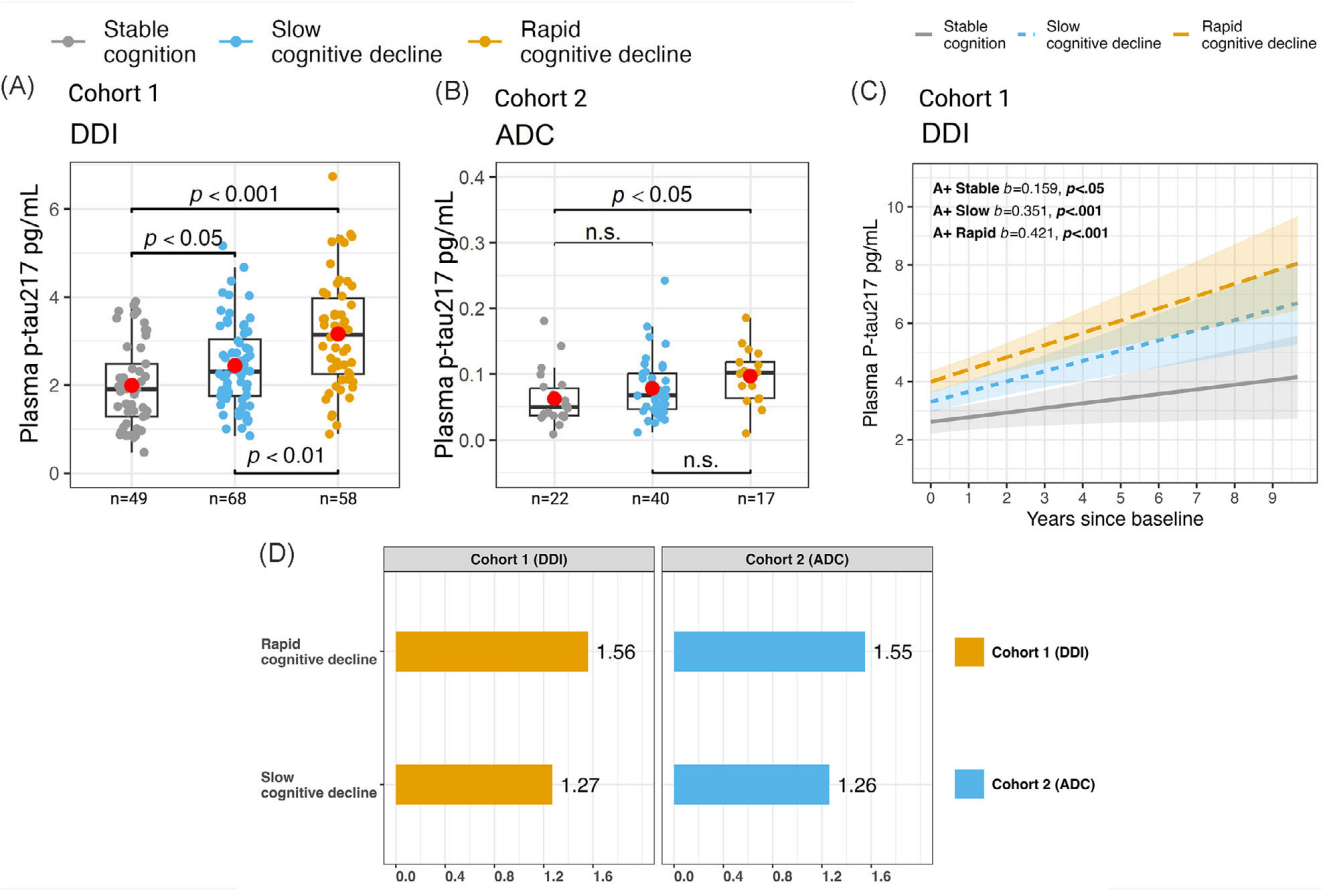
Between cluster comparison of plasma phosphorylated tau‐217 (p‐tau217) in Cohort 1 and Cohort 2. (A) Cohort 1 and (B) Cohort 2 show between‐cluster comparisons of plasma p‐tau217, with red dots indicating means. Cohort 1 utilized the University of Gothenburg in‐house p‐tau217 assay, whereas Cohort 2 utilized a commercially available kit by Janssen. Both used Quanterix Simoa HD‐X setups. All statistical tests were two‐sided and false‐discovery rate was used to adjust for multiple comparisons in each cohort. Due to differing assays and the range of pg/mL between assays, (D) shows the similarities of mean fold changes for cognitive decline clusters as compared to the stable cognition clusters in both cohorts. (C) Shows steeper plasma p‐tau217 increases over time in slow‐ and rapid‐decline clusters compared to the stable cognition cluster, with 95% confidence intervals. Unstandardized beta coefficients (b) illustrate individual slopes for each cluster. Created in BioRender. Kirsebom, B. (2025) https://BioRender.com/o54z804.

In Cohort 1 (Figure 2A), plasma p‐tau217 levels showed a stepwise elevation across clusters, with significantly higher levels in the slow cognitive decline cluster (*b* = 0.67, mean fold change = 1.27, *p* < 0.05) and the rapid cognitive decline cluster (*b* = 1.38, mean fold change = 1.56, *p* < 0.001) compared to the stable cluster. Similarly, in Cohort 2 (Figure 2B), plasma p‐tau217 levels also showed stepwise elevations, with higher levels in the slow cognitive decline cluster (*b* = 0.016, mean fold change = 1.25, *p* = 0.169) and the rapid cognitive decline cluster (*b* = 0.034, mean fold change = 1.55, *p* < 0.05). Although only Cohort 1 showed statistically significant differences in both decline clusters, the mean fold changes were consistent across cohorts, indicating similar trends in p‐tau217 elevation regardless of assay differences (Figure 2D). To assess whether cluster membership, rather than baseline diagnostic status, was associated with plasma p‐tau217 levels, we conducted additional analyses in Cohort 1. Specifically, we used a linear regression model to compare baseline plasma p‐tau217 concentrations between cognitive decline clusters, including clinical diagnosis (CN/SCD vs MCI) as a covariate. The results were consistent with previous findings: higher p‐tau217 levels were observed in the slow cognitive decline cluster (*b* = 0.63, *p* < 0.05) and the rapid decline cluster (*b* = 1.29, *p* < 0.001) compared to the stable cluster. Baseline diagnosis was not a significant covariate (*b* = 0.19, *p* = 0.395). We then conducted analysis of variance (ANOVA) to compare baseline plasma p‐tau217 concentrations between CN/SCD and MCI cases within each cognitive decline cluster (see Figure S3). After adjusting for multiple comparisons (FDR), no significant differences in plasma p‐tau217 levels were observed between CN/SCD and MCI cases within the stable cognition (*p* = 0.571) and slow cognitive decline clusters (*p* = 0.595). Within the rapid cognitive decline cluster, MCI cases exhibited higher mean plasma p‐tau217 levels than CN/SCD cases; however, this difference did not reach statistical significance (*p* = 0.099), and the CN/SCD subgroup was small (*n* = 9), likely limiting the reliability of this comparison. In Cohort 2, the number of cases stratified by both cluster and diagnosis was insufficient for meaningful statistical analysis.[Fig alz70319-fig-0001], [Table alz70319-tbl-0002]

### 3.4 Steeper plasma p‐tau217 increase over time in clinical progressors

Cohort 1 included longitudinal plasma p‐tau217 measurements for 185 participants (99.46%) represented in the clusters, with 438 observations over up to 9.66 years from baseline (mean = 3.75 years, SD = 1.74). Although all clusters showed significant increases in plasma p‐tau217 over time (Figure 2C), the increases were steeper in the slow cognitive decline (*b* = 0.351, *SE* = 0.07, *p* < 0.001) and rapid cognitive decline (*b* = 0.421, *SE* = 0.09, *p* < 0.001) clusters compared to the stable cognition cluster (*b* = 0.159, *SE* = 0.07, *p* < 0.05). However, only the rapid decline group exhibited a significantly steeper increase than the stable group (*b* = 0.262, SE = 0.11, *p* < 0.05), whereas the slow decline group showed a trend‐level significance (*b* = 0.192, SE = 0.10, *p* = 0.06).

## 4 DISCUSSION

In this study, we assessed the heterogeneity of clinical progression in Aβ+ cognitively normal and MCI individuals across two independent cohorts. Using a data‐driven k‐means clustering approach to analyze individual trajectories of cognitive change over time, we identified three clusters of cognitive change in both cohorts: one representing stable cognition with no or minimal cognitive change over time, and two representing either slower or more rapid cognitive decline. Furthermore, in both cohorts, a stepwise elevation in baseline plasma p‐tau217 levels was observed in the clusters with cognitive decline compared to those with stable cognition. In Cohort 1, repeated plasma p‐tau217 measurements demonstrated a progressive increase in plasma p‐tau217 concentrations over time, corresponding with clinical severity, particularly in the rapid cognitive decline cluster.

Higher plasma p‐tau217 levels have been shown to align with degree of tau‐tangle burden,^24^ clinical severity, and future cognitive decline.^19^, ^25^, ^26^ Our findings further validate these observations and extend them by emphasizing the importance of accounting for heterogeneity of clinical progression in non‐demented AD cases, beyond conventional criteria for cognitive normalcy and MCI.^13^, ^14^ Notably, 15% of individuals in Cohort 1 and 26.4% in the Cohort 2, initially classified as cognitively normal, were in the rapid cognitive decline cluster, likely representing cases with a more aggressive disease course. This highlights the limitations of relying solely on cognitive stage and amyloid status to assess disease severity and progression risk. In contrast, a substantial proportion of MCI cases were in the cognitively stable cluster (39.3% in Cohort 1 and 54.3% in Cohort 2). Although MCI is a well‐established risk factor for cognitive decline,^13^ these findings suggest that many individuals remain stable or progress slowly. This aligns with evidence that not all Aβ+ individuals develop clinical symptoms during their lifetime.^11^ Moreover, it raises an important question: is defining AD solely based on biomarker evidence of amyloid plaques an adequate criterion for initiating disease‐modifying treatments? Our cognitive decline clusters included more MCI cases, which showed worse baseline cognitive performance than the stable cluster. This is partially expected, as these individuals are likely further along in the disease course rather than solely representing cases of rapid progression during the observation period. Nonetheless, this highlights the heterogeneity of progression, even among Aβ+ individuals with MCI.

Higher baseline plasma p‐tau217 concentrations in individuals poised for rapid cognitive decline suggest that this biomarker tracks disease severity, irrespective of cognitive stage. Moreover, clinical progressors exhibit steeper increases in plasma p‐tau217 levels over time, whereas cognitively stable cases show modest changes, reinforcing the biomarker's potential utility for disease monitoring.^27^ Plasma p‐tau217 has been extensively studied as a blood‐based diagnostic tool for AD,^19^, ^20^, ^21^ but its precision in Aβ+ cognitively normal individuals is lower due to reduced positive predictive values (PPVs) in this group.^19^, ^28^ This can increase false positives, which is expected in general population studies with lower pre‐test probabilities of amyloid pathology.^41^ However, in studies using gold standard methods like CSF or amyloid PET, where amyloid status is well defined, this limitation should be less pronounced. It is important to note that our data‐driven analysis shows that not all cognitively normal individuals with amyloid pathology progress clinically over a 10‐year period, highlighting the heterogeneity among Aβ+ individuals. This underscores the importance of markers like plasma p‐tau217 that reflect the biological severity of AD rather than the mere presence of amyloid pathology. Plasma p‐tau217 levels rise with increasing clinical severity, suggesting it serves as a dynamic indicator of disease progression. The lower PPV in CN individuals may reflect smaller p‐tau217 elevations in earlier disease stages, highlighting its sensitivity to disease trajectory over static amyloid status. Aβ+ CN participants with higher plasma p‐tau217 concentrations may face greater risk of clinical progression. Two cutoff approaches, designed to minimize diagnostic discrepancies with CSF or amyloid PET,^41^ could also help identify those at higher progression risk, offering a refined method for stratifying predementia AD populations.

Interestingly, plasma p‐tau217 reductions have been observed following treatment, suggesting a disease‐modifying effect downstream of Aβ plaque removal.^4^ However, for participants with lower disease burden, the extent to which p‐tau217 levels may decrease remains unclear. The donanemab Phase 3 trial demonstrated more pronounced slowing of progression in individuals with lower tau‐tangle burden compared to the overall trial population.^2^ Although data for patients with higher tau‐tangle burden have not yet been published, it is plausible that earlier treatment in the disease course could yield greater benefits. Nevertheless, the heterogeneity of clinical progression was not accounted for in anti‐amyloid mAb trial designs^1^, ^2^ raising the possibility that treatment effects may have been obscured or biased. As we have shown, heterogeneity in clinical progression among cognitively normal and MCI cases is likely tied to underlying disease severity. Although anti‐amyloid treatments may be more effective at earlier stages,^12^ identifying patients most likely to benefit is essential. Beyond its diagnostic utility, plasma p‐tau217 could stratify patients by progression risk, guide trial participant selection, and improve detection of treatment effects. Longitudinal plasma p‐tau217 measurements could serve as secondary endpoints to monitor treatment efficacy and align biomarker changes with clinical outcomes. Repeated blood sampling could also track disease progression over time, enabling more precise evaluation of therapeutic benefits.

We excluded demographics such as age, sex, and education from our main cognitive models, focusing instead on individual trajectories of cognitive change. Although these factors influence cognitive performance in normative populations,^15^, ^16^ they are also associated with AD risk.^39^, ^40^ In our study, all participants had established amyloid pathology, and importantly, no significant demographic differences were observed between clusters. This suggests that individual trajectories of cognitive decline in AD may, to some extent, be independent of demographic factors. Supporting this, our sensitivity analyses showed that when demographics were included in the models, the resulting clusters exhibited almost perfect agreement with those from the original analysis.

Here, we showed that higher plasma p‐tau217 levels are associated with clinical severity and increased risk of clinical progression, reinforcing its role as a core AD biomarker. However, plasma p‐tau217 accounts for only part of the variance in clinical progression. Factors such as genetic predisposition^6^ and co‐pathologies^7^ likely influence progression rates and responses to treatments. Future research should explore the impact of co‐pathologies as accessible biomarkers become available. In addition, cognitive stability despite Aβ biomarker evidence suggests the presence of protective factors, whether genetic or environmental. Identifying these factors requires large‐scale studies, which could inform prevention strategies and novel therapeutic approaches.

This work is subject to some limitations. First, although the cognitive composite likely reduces within‐subject variability, it may obscure domain‐specific deficits sensitive to early AD or the stages of cognitive decline over time. Our k‐means clustering approach is one method to assess heterogeneity, but more advanced techniques, such as machine learning, could offer further refinement. Although our findings suggest that elevated p‐tau217 is associated with cognitive decline trajectories beyond diagnostic status alone, the small sample size of CN cases within the rapid cognitive decline cluster limits the robustness of this analysis and warrants cautious interpretation. Larger samples of Aβ+ CN individuals are needed to confirm this observation. Finally, although we replicated baseline differences in plasma p‐tau217 across cohorts using different assays, the lower availability of this marker in the ADC cohort reduced statistical power. Further validation in independent cohorts is needed to confirm these findings.

In conclusion, this study underscores the potential of plasma p‐tau217 as an objective measurement for tracking clinical heterogeneity and progression in AD. The agreements between plasma p‐tau217 increases and clinical severity might guide patient selection and stratification in clinical practice and trial selection. Future research should aim to validate these results in larger cohorts and explore the mechanisms underlying cognitive stability despite biomarker evidence of AD pathology to inform novel therapeutic strategies.

## CONFLICT OF INTEREST STATEMENT

B. E. K. has served as a consultant for Biogen and medical advisory boards for Biogen and Eli Lilly. H. Z. has served at scientific advisory boards and/or as a consultant for Abbvie, Acumen, Alector, Alzinova, ALZpath, Amylyx, Annexon, Apellis, Artery Therapeutics, AZTherapies, Cognito Therapeutics, CogRx, Denali, Eisai, Enigma, LabCorp, Merry Life, Nervgen, Novo Nordisk, Optoceutics, Passage Bio, Pinteon Therapeutics, Prothena, Quanterix, Red Abbey Labs, reMYND, Roche, Samumed, Siemens Healthineers, Triplet Therapeutics, and Wave; has given lectures sponsored by Alzecure, BioArctic, Biogen, Cellectricon, Fujirebio, Lilly, Novo Nordisk, Roche, and WebMD; and is a co‐founder of Brain Biomarker Solutions in Gothenburg AB (BBS), which is a part of the GU Ventures Incubator Program (outside submitted work). K.B. has served as a consultant and at advisory boards for Abbvie, AC Immune, ALZPath, AriBio, Beckman‐Coulter, BioArctic, Biogen, Eisai, Lilly, Moleac Pte. Ltd, Neurimmune, Novartis, Ono Pharma, Prothena, Quanterix, Roche Diagnostics, Sanofi and Siemens Healthineers; has served at data monitoring committees for Julius Clinical and Novartis; has given lectures, produced educational materials and participated in educational programs for AC Immune, Biogen, Celdara Medical, Eisai and Roche Diagnostics; and is a co‐founder of Brain Biomarker Solutions in Gothenburg AB (BBS), which is a part of the GU Ventures Incubator Program, outside the work presented in this article. T.F. has served as a consultant and on advisory boards for Biogen, Eisai, Novo Nordisk, Eli Lilly, and Roche. R.E.S. has served on advisory boards for Eisai and Eli Lilly. A.V.H. has participated in educational programs for Eisai and an educational advisory board for Lilly. The remainder of the authors report no relevant conflict of interest relevant for this publication. Author disclosures are available in the Supporting Information.

## CONSENT STATEMENT

The Dementia Disease Initiation study was approved by the Regional Committees for Medical and Health Research Ethics in Norway and conducted in line with the guidelines provided by the Declaration of Helsinki and the Norwegian Health and Research act. All participants gave written informed consent before participating in the study. The Amsterdam Dementia Cohort study was approved by the ethics committee of the Amsterdam UMC and the Biobank Research Ethics Committee of the Amsterdam University Medical Center (location Vrije Universiteit). All patients gave written informed consent, and the study was conducted in accordance with the guidelines provided by the Declaration of Helsinki.

## Supporting information



Supporting Information

Supporting Information
