# Supplementary material for: Repeated plasma p‐tau217 measurements to monitor clinical progression heterogeneity
Source: Alzheimers Dement. 2025 May 29;21(5):e70319. doi: 10.1002/alz.70319 (PMC12122256; doi:10.1002/alz.70319)
Supplement: Supplementary file 2 — Supporting Information [file ALZ-21-e70319-s001.docx]

**Supplementary material.**Supplementary Table 1 presents linear mixed-effects models (LMMs) assessing longitudinal changes in each cognitive test evaluated in Cohort 1 (Dementia Disease Initiation), along with the cognitive composite score, which includes the Trail Making Test-B (TMT-B) and the Consortium to Establish a Registry for Alzheimer's Disease (CERAD) word list delayed memory recall. Marginal *R²* effect sizes, illustrating the fixed effects of time on cognitive change, were computed using the “performance” R package. Supplementary Figures 1A and 1B display elbow plots used to visually determine the optimal number of clusters for extraction from the k-means clustering models in each cohort.

| **Supplementary table 1.** Cognitive test selection for cognitive composite | | | | | | |
| --- | --- | --- | --- | --- | --- | --- |
|  | Parameter | *b* | *SE* | *t* | *p* | *Marginal R^2^* |
| COWAT/FAS | Intercept | 38.21 | 0.87 | 43.88 | < .001 |  |
|  | Years | -0.29 | 0.18 | -1.64 | n.s. | 0.003 |
|  |  |  |  |  |  |  |
| VOSP Silhouettes | Intercept | 21.23 | 0.32 | 65.47 | < .001 |  |
|  | Years | -0.14 | 0.07 | -2.01 | <.05 | 0.004 |
|  |  |  |  |  |  |  |
| CERAD memory recall | Intercept | 4.97 | 0.21 | 23.28 | < .001 |  |
|  | Years | -0.23 | 0.04 | -5.42 | <.001 | 0.025 |
|  |  |  |  |  |  |  |
| TMT-B | Intercept | 4.67 | 0.03 | 137.12 | < .001 |  |
|  | Years | 0.04 | 0.01 | 5.41 | <.001 | 0.029 |
|  |  |  |  |  |  |  |
| Cognitive composite | Intercept | 0.52 | 0.01 | 38.30 | < .001 |  |
|  | Years | -0.02 | 0.01 | -7.18 | <.001 | 0.048 |
| *Abbreviations:* *b,* unstandardized beta coefficient; SE, standard error of the unstandardized beta coefficient; t, t-test; p, p-value: R^2^, R-squared.  *Notes:*  The marginal R^2^ is a measure of the overall explained variance of change in the cognitive test over time. The table shows each model arranged from the least to the most explained variance. Here, TMT-B and CERAD memory recall are selected for the cognitive composite. | | | | | | |

*
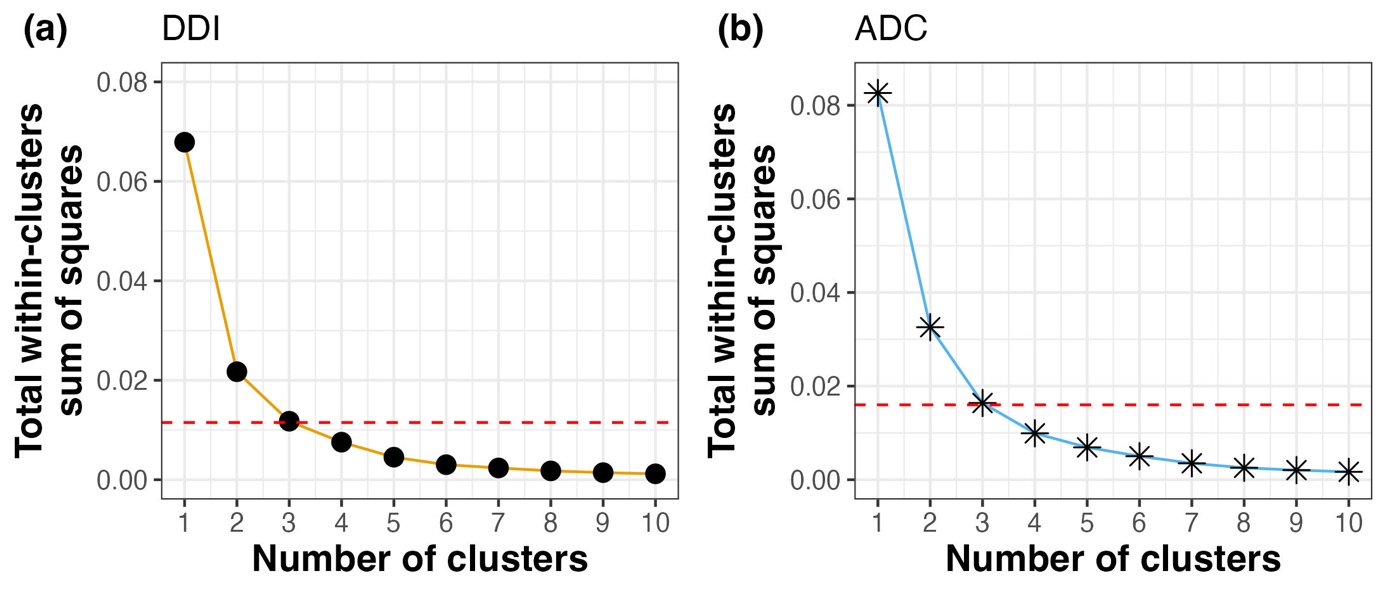
*

**Supplementary figure 1.** Elbow plots used to select the number of clusters following k-means clustering of individual slopes of cognitive change in cohort 1 (a) and cohort 2 (b). The red dashed horizontal lines shows the decision to select three clusters in both cohorts.

| **Supplementary Table 2.** Between-cluster comparisons of baseline demographics (age, sex, and years of education) for clusters derived from cognitive trajectories adjusted for demographics, in the Dementia Disease Initiation (DDI) cohort and the Amsterdam Dementia Cohort (ADC) | | | | |
| --- | --- | --- | --- | --- |
|  | **Cohort 1:** **Dementia Disease Initation cohort** | | | |
|  | **Stable  cognition 56** | **Slow cognitive decline  72** | **Rapid  cognitive decline 58** | **Slow vs rapid   cognitive decline** |
| **Age** Mean (SD) | 66.54  (8.43) | 68.33^n.s.^ (6.56) | 68.97^n.s.^ (7.58) | ^n.s.^ |
| **Years of education** Mean (SD) | 13.96 (3.17) | 13.53^n.s.^ (3.27) | 13.53^n.s.^ (2.91) | ^n.s.^ |
| **Female** n (%) | 26  (46.4) | 44 (61.1) | 29 (50.0) | ^n.s.^ |
|  | **Cohort 2: Amsterdam Dementia Cohort** | | | |
|  | **Stable  cognition 50** | **Slow cognitive decline  110** | **Rapid  cognitive decline 47** | **Slow vs rapid   cognitive decline** |
| **Age** Mean (SD) | 65.06 (7.10) | 66.25^n.s.^ (6.43) | 65.94^n.s.^ (7.17) | ^n.s.^ |
| **Years of education** Mean (SD) | 12.54 (3.11) | 12.07^n.s.^ (3.00) | 12.77^n.s.^ (3.39) | ^n.s.^ |
| **Female** n (%) | 23 (46.0%) | 52 (47.3%) | 25 (46.8) | ^n.s.^ |
| Abbreviations: SD, standard deviation; n, number of cases; %, percentage; n.s. = non-significant; *, <.05,**, <.01, ***<.001 as compared with the stable cognition group, if not otherwise specified. | | | | |


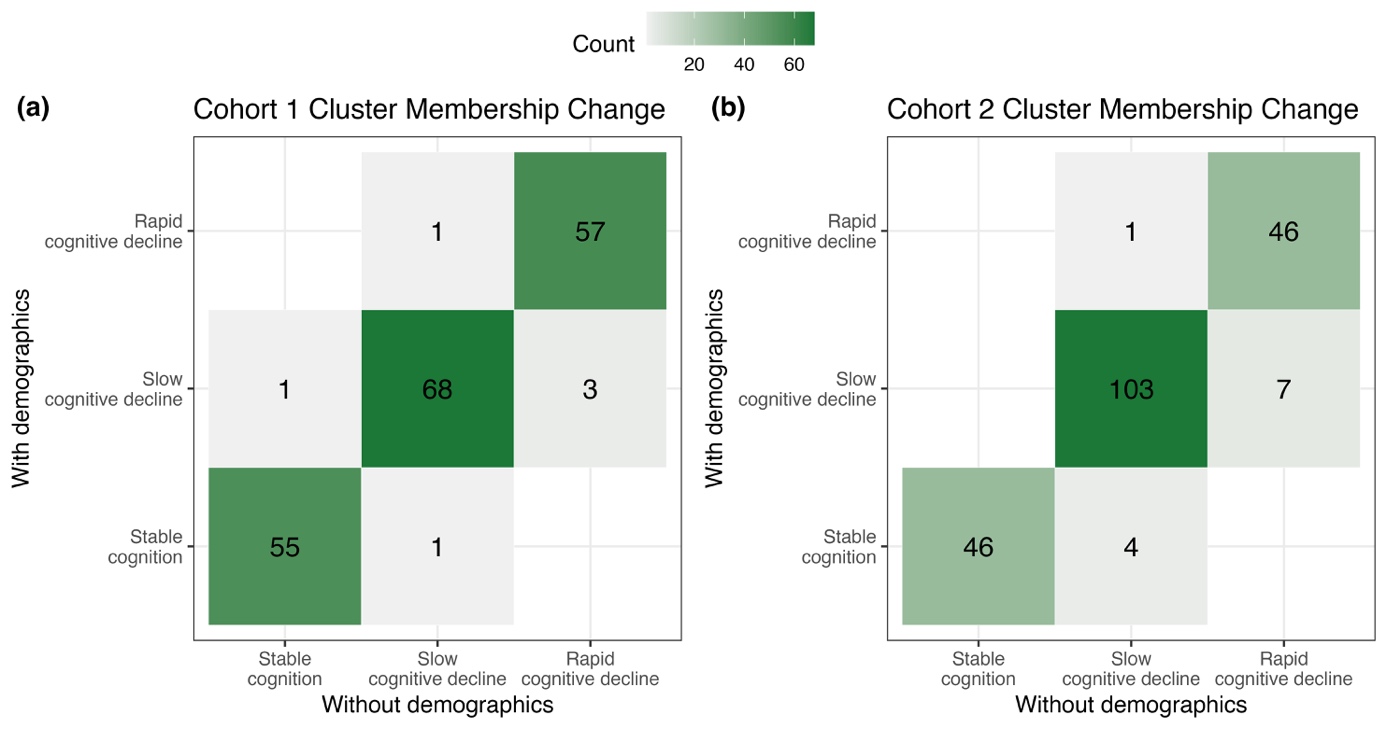


**Supplementary figure 2.** Heatmap showing changes in cluster membership between clusters derived with and without adjustments for demographics (age, sex and years of education). Panel (a) shows cohort 1 (Dementia Disease Initiation, k = 0.951, z = 18.3, p<.001), and panel (b) shows cohort 2 (Amsterdam Dementia Cohort, k = 0.905, z = 18.0, p<.001) both demonstrating minimal changes in cluster membership and high agreement between methods.


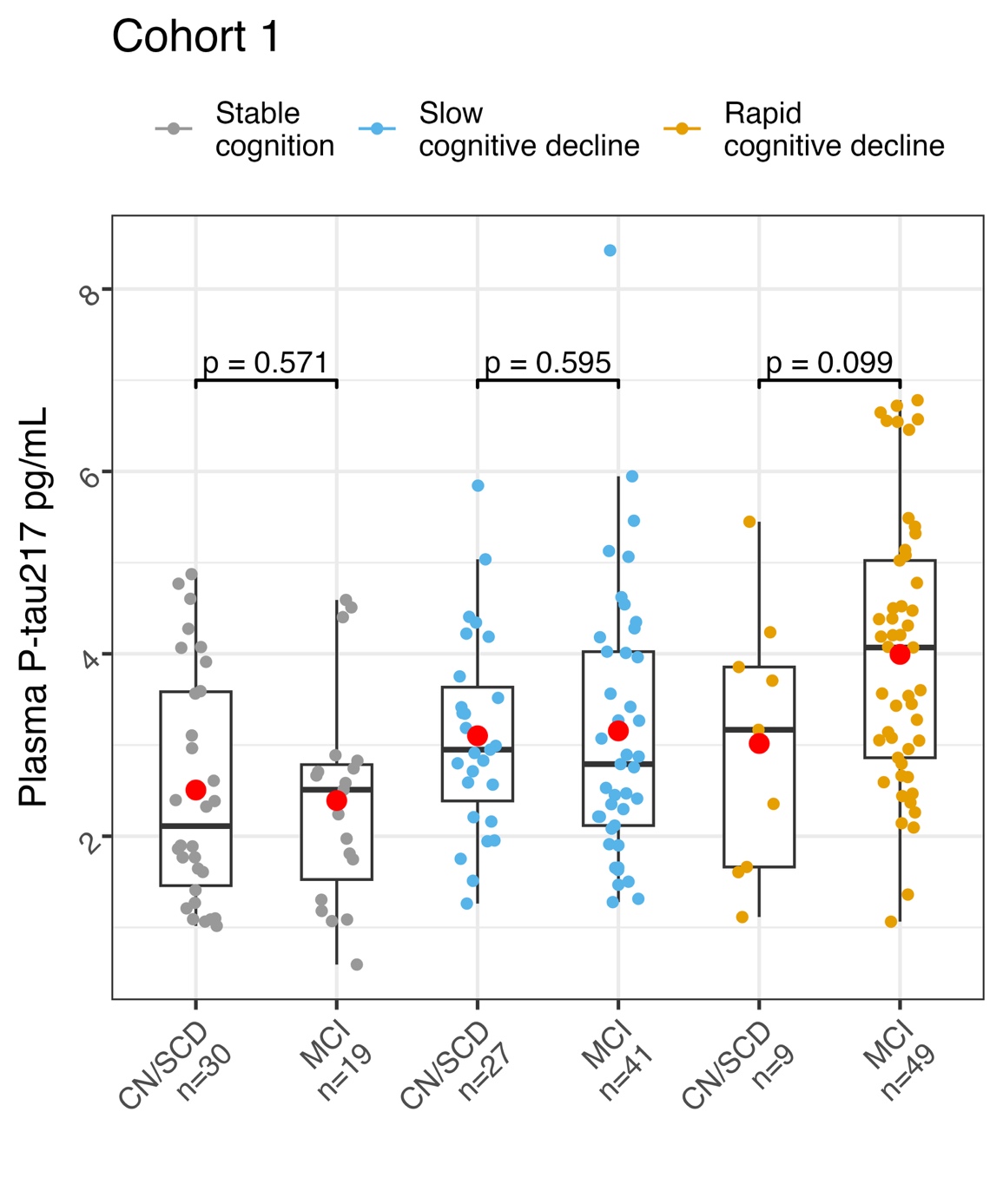


**Supplementary Figure 3.** Comparison of baseline plasma p-tau217 levels between diagnostic groups (CN/SCD vs. MCI) within each cognitive decline cluster, performed in Cohort 1 (Dementia Disease Initiation). Horizontal brackets indicate False Discovery Rate (FDR)-adjusted post-hoc comparisons following Analysis of Variance (ANOVA).
